# Bias-dependent photoresponsivity of multi-layer MoS_2_ phototransistors

**DOI:** 10.1186/s11671-017-2368-2

**Published:** 2017-11-21

**Authors:** Jinwu Park, Youngseo Park, Geonwook Yoo, Junseok Heo

**Affiliations:** 1 Department of Electrical and Computer Engineering, Ajou University, Suwon, 16499 South Korea; 2 School of Electronic Engineering, Soongsil University, Seoul, 06938 South Korea

## Abstract

We studied the variation of photoresponsivity in multi-layer MoS_2_ phototransistors as the applied bias changes. The photoresponse gain is attained when the photogenerated holes trapped in the MoS_2_ attract electrons from the source. Thus, the photoresponsivity can be controlled by the gate or drain bias. When the gate bias is below the threshold voltage, a small amount of electrons are diffused into the channel, due to large barrier between MoS_2_ and source electrode. In this regime, as the gate or drain bias increases, the barrier between the MoS_2_ channel and the source becomes lower and the number of electrons injected into the channel exponentially increases, resulting in an exponential increase in photoresponsivity. On the other hand, if the gate bias is above the threshold voltage, the photoresponsivity is affected by the carrier velocity rather than the barrier height because the drain current is limited by the carrier drift velocity. Hence, with an increase in drain bias, the carrier velocity increases linearly and becomes saturated due to carrier velocity saturation, and therefore, the photoresponsivity also increases linearly and becomes saturated.

## Background

Recently, transition metal dichalcogenide (TMD) materials including molybdenum disulfide (MoS_2_) and tungsten diselenide (WSe_2_) have received considerable attention as the channel material for next generation nanoelectronic devices [1–6]. In particular, thin-film transistors that use MoS_2_ exhibit interesting electric characteristics such as high electron mobility (~ 200 cm^2^ V^−1^ s^−1^), high current ON/OFF ratio (~ 10^8^), and low subthreshold swing (~ 70 mV dec^−1^) in a single-layer MoS_2_ transistor [7]. In addition, MoS_2_ is attracting attention as a light absorbing layer in optoelectronic devices because of its bandgap energy (single-layer MoS_2_ has a direct bandgap of 1.8 eV [8] and bulk MoS_2_ has an indirect bandgap of 1.2 eV [9]) and large absorption coefficient (*α* = 1–1.5 × 10^6^ cm^−1^ for single-layer [10] and 0.1–0.6 × 10^6^ cm^−1^ for bulk [11]). Hence, phototransistors using MoS_2_ have a low dark current in the OFF state and high photoresponsivity. The performance of MoS_2_ phototransistors have been improved by introducing an additional layer such as graphene [12–15], quantum dot [16–18], organic dye [19], WS_2_ [20–22], ZnO [23], and p-type MoS_2_ [24] or by changing the gate dielectric [7, 25, 26]. In this way, many studies have been actively conducted to improve the photoresponsivity through additional manufacturing processes; however, there is a lack of research on the gain control and specific understanding of MoS_2_ phototransistors. When gain control is enabled, a wide range of light intensities can be reliably detected, and the gain can be increased without any additional manufacturing process. In this context, we investigated the bias (drain or gate)-controlled photoresponsivity in multi-layer MoS_2_ phototransistors.

## Methods

**Fig. 1:**
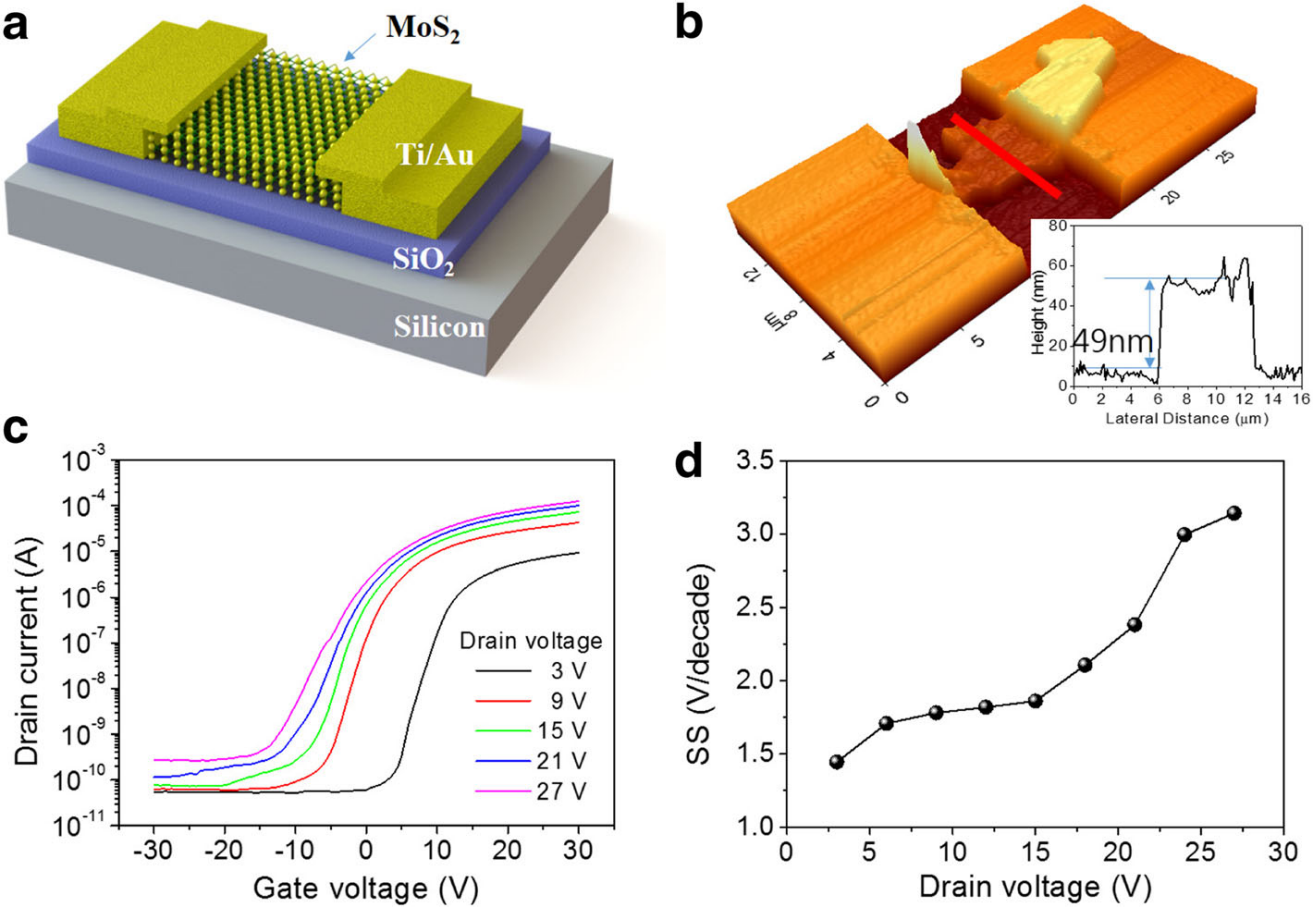
The fabricated MoS_2_ phototransistor and electrical characteristics. **a** Schematic diagram of the fabricated multi-layer MoS_2_ phototransistor. **b** Atomic Force Microscope (AFM) image of the phototransistor. The inset is the cross-section plot along the red line in the AFM image. **c** Transfer characteristics of the multi-layer MoS_2_ phototransistor with the drain voltages of 3, 9, 15, 21, and 27 V in the dark. **d** Variations in the subthreshold swing with increasing drain bias

Figure 1a shows the schematic diagram of the fabricated multi-layer MoS_2_ phototransistor. We grew the 250 nm SiO_2_ on a heavily n-doped silicon substrate. The multi-layer MoS_2_ flakes were mechanically exfoliated from bulk MoS_2_ (Graphene Supermarket, USA) and transferred to a SiO_2_/Si substrate by using the conventional scotch-tape method [27]. The source and drain electrodes were patterned by photo-lithography and Ti/Au (5/80 nm) were deposited on the patterned by using an e-beam evaporator. Figure 1b shows the AFM (Atomic Force Microscope) image of the fabricated phototransistor. The channel length and width are 7.31 and 4.74 μm, respectively, and the inset shows the thickness of the multi-layer MoS_2_ is approximately 49 nm, which corresponds to about 75 layers, assuming the thickness of one layer to be 0.65 nm [28, 29].

## Results and discussion

Figure 1c shows the transfer characteristics of the multi-layer MoS_2_ phototransistor with drain biases of 3, 9, 15, 21, and 27 V in the dark. The current–voltage characteristics of the fabricated multi-layer MoS_2_ phototransistor were measured using a dual-channel source meter (Keithley 2614B) at room temperature and N_2_ ambient. The ON/OFF ratio is approximately 10^5^. The field effect mobility was estimated to be 18.6 cm^2^/V s for a drain bias of 3 V from the following equation [26]:

1 $$ {\mu}_{\mathrm{eff}}=\left({g}_m\cdot L\ \right)/\left(\ {C}_{\mathrm{OX}}\cdot W\cdot {V}_{\mathrm{DS}}\right) $$

where *L* is the channel length, *W* is the channel width, and the oxide capacitance *C*_OX_ is 1.38 × 10^−8^ F/cm^2^. It was clearly observed that when the drain bias is increased, the threshold voltage decreases and the subthreshold swing increases. This indicates that the threshold voltage and subthreshold swing are affected by the drain bias. In general, the threshold voltage is estimated by the equation:

2 $$ {V}_{\mathrm{th}}={V}_{\mathrm{GS}}(0)-{V}_{\mathrm{DS}}/2 $$

where *V*_GS_(0) is the intercept between the trend line in a linear part of the transfer curve and the *x*-axis. However, Eq. (2) assumes a small drain bias such that the velocity saturation effects are negligible (*V*_DS_〈〈*L* ⋅ *ν*_sat_/*μ*_eff_ = 10 V, where *ν*_sat_ is the saturation velocity and *μ*_eff_ is the field effect mobility); therefore, it is difficult to extract the exact threshold voltage for a large drain bias. For this reason, we extracted only the change in subthreshold swing and confirmed the effect of the drain bias on the channel. Figure 1d shows the change in subthreshold swing extracted from the slope of the linear part of the log(*I*_*D*_) − (*V*_GS_) graph for different drain biases. The subthreshold swing increased from 1.44 V/decade to 3.14 V/decade when the drain bias increased from 3 to 27 V. This implies that a large drain bias lowers the barrier between the MoS_2_ channel and the Au source electrode, thus weakening the channel controllability of the gate bias.

**Fig. 2:**
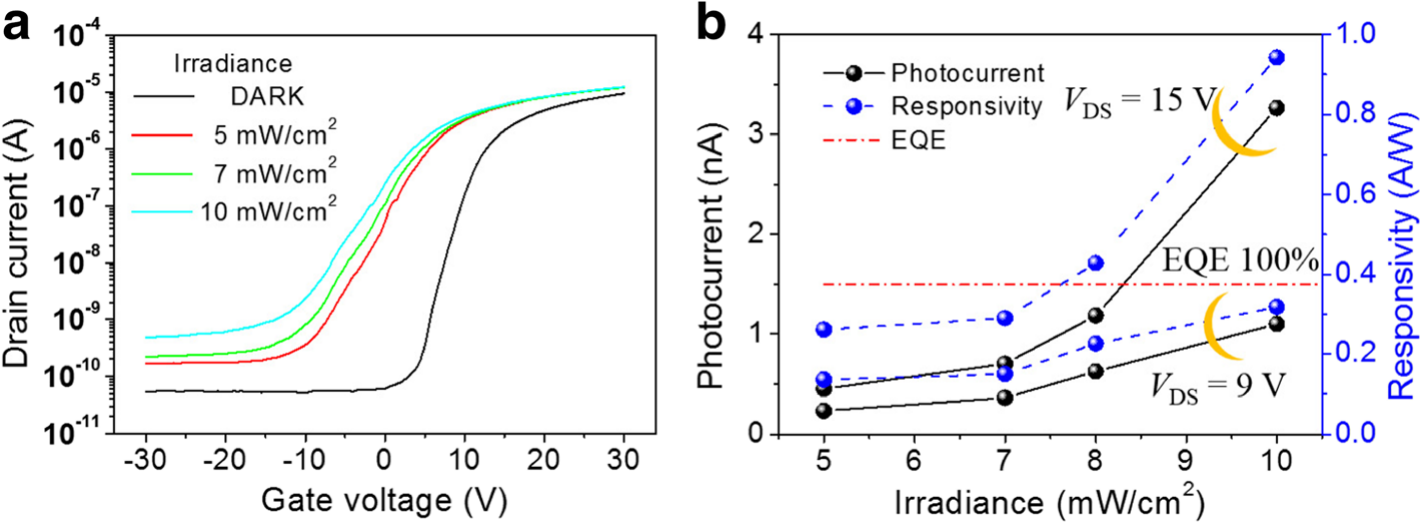
Photoresponse characteristics of MoS_2_ phototransistors depending on illuminated light intensity. **a** Transfer characteristics with a constant *V*_DS_ = 3 V under illumination with three different intensities of light (5, 7, and 10 mW/cm^2^). **b** Change in photocurrent with increase in intensity of light when different drain biases (*V*_DS_ = 9, 15 V) and a constant gate bias (*V*_GS_ = − 30 V) are applied

To investigate the responsivity of the MoS_2_ phototransistor, we measured the transfer characteristics at various illumination power densities using a 466-nm wavelength diode-pumped solid-state (DPSS) laser. Figure 2a shows the transfer characteristics of the multi-layer MoS_2_ phototransistor under dark and under three different light intensities (5, 7, and 10 mW/cm^2^), at a drain voltage of 3 V. As the light intensity increases, the transfer curve shifts to the left, which shows that the photogenerated holes are trapped in the MoS_2_ channel and act as a positive gate bias [13, 30, 31]. Figure 2b shows that the variation of photocurrent and responsivity when the light intensity and drain bias increase at a constant gate bias of − 30 V. The photocurrent is obtained by the difference between the drain current under illumination and in the dark (*I*_ph_ = *I*_illuminated_ − *I*_dark_), and the responsivity is defined by *I*_ph_/*P*_light_, where *I*_ph_ is the photocurrent and *P*_light_ is the optical power illuminated on the MoS_2_ channel. As the drain bias and light intensity increase, the photocurrent and responsivity increase. Considering a laser with a wavelength of 466 nm, the responsivity corresponding to 100% of the external quantum efficiency (EQE) is 0.375 A/W, and the measured responsivity exceeds this value, when the drain bias is 15 V and the light intensity is 8 mW/cm^2^. This means that there is a photoresponse gain in this multi-layer MoS_2_ phototransistor and that it is affected by the drain bias.

**Fig. 3:**
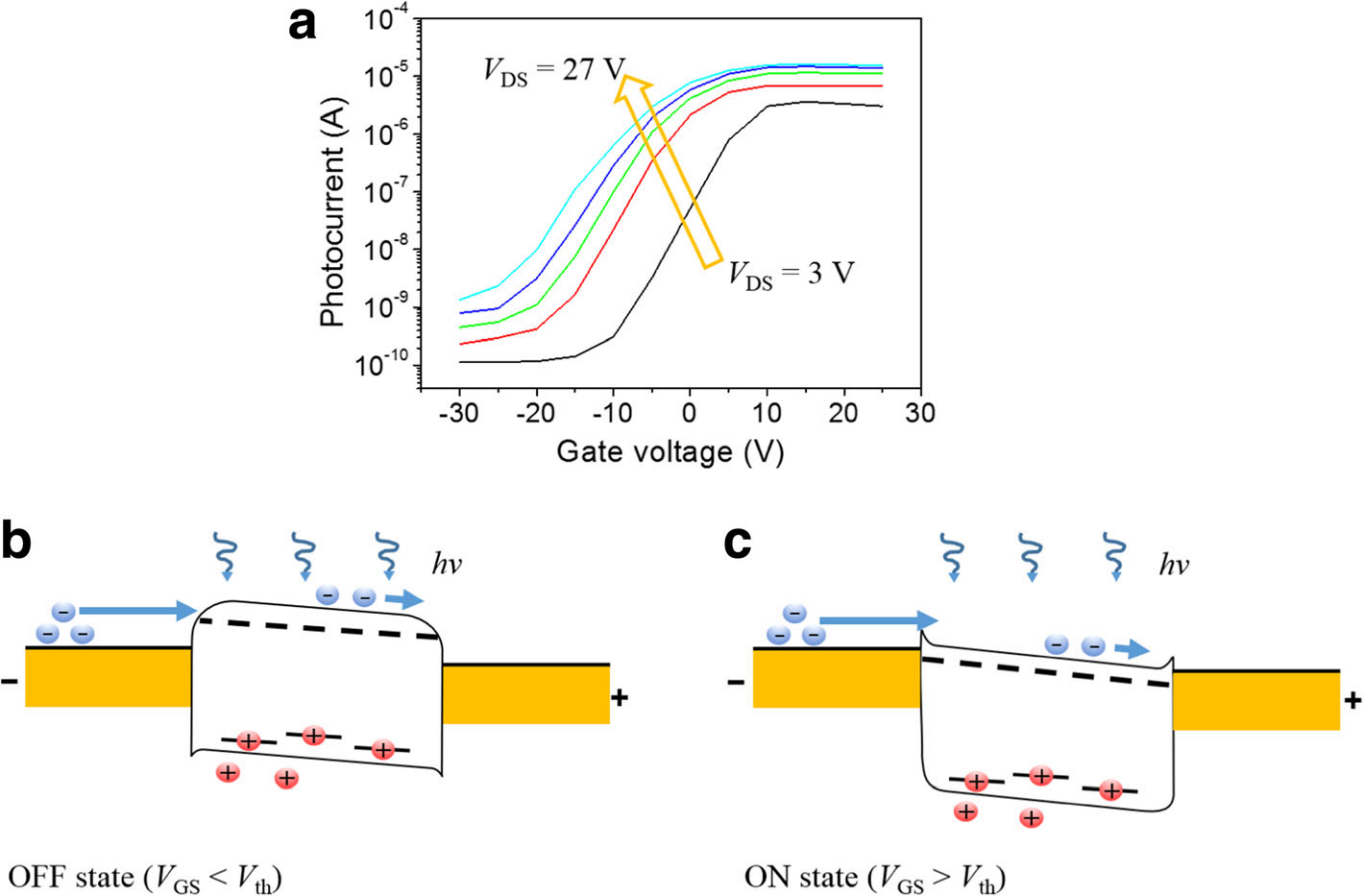
Photoresponse of MoS_2_ phototransistors depending on applied bias. **a** Photocurrent at various drain biases (3, 9, 15, 21, and 27 V) and a constant light intensity (5 mW/cm^2^) depending on the gate bias. **b, c** The energy band diagrams of a multi-layer MoS_2_ phototransistor

In order to observe the change in photoresponsivity according to the gate voltage, we measured the photocurrent while increasing the drain voltage from 3 to 27 V under 5 mW/cm^2^ light illumination (Fig. 3a). As the applied gate bias increases, the photocurrent increases exponentially in the OFF state (*V*_GS_ < *V*_th_) and becomes saturated in the ON state (*V*_th_ < *V*_GS_). This is because, when the applied gate bias is − 30 V (OFF state) and it is illuminated (Fig. 3b), a large barrier is formed between the MoS_2_ channel and the source/drain (Au) electrodes. Thus, the electrons needed to maintain the channel neutrality, which was destroyed by the trapped holes, are not well injected into the channel. However, as the gate bias increases up to the threshold voltage, the barrier becomes smaller and the electrons can easily diffuse into the MoS_2_ channel. Therefore, the photocurrent increases exponentially before the threshold voltage. On the other hand, if the gate bias becomes larger than the threshold voltage, that is, when the device is turned ON, the barrier is sufficiently lowered and the photocurrent is saturated (Fig. 3c). It was also noticed that the photocurrent increases in both the OFF and ON states as the drain bias increases. This means that unlike the photoresponse properties of the conventional phototransistor, which is measured only in the OFF state [26, 32], there is photoresponse gain even in the ON state as the drain voltage increases.

**Fig. 4:**
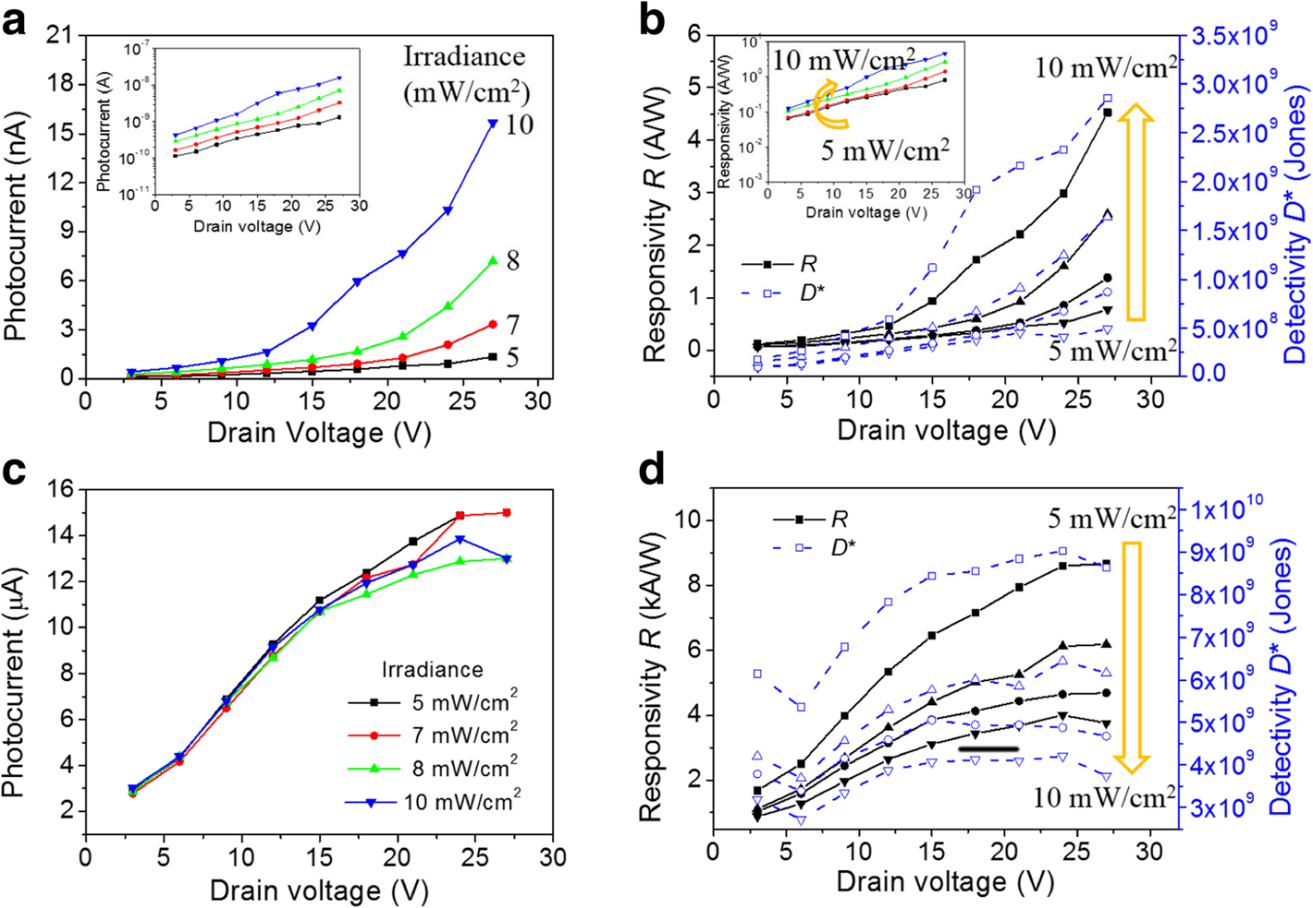
Photoresponse characteristics measured at four different irradiances (5, 7, 8, and 10 mW/cm^2^) when the drain bias is increased. **a** Photocurrent, **b** responsivity, and specific detectivity in the OFF state. Insets in **a** and **b** are plotted with the log scale of the photocurrent and responsivity, respectively. **c** Photocurrent, **d** responsivity, and specific detectivity in the ON state

In order to verify the effect of drain bias on the photoresponsivity of the MoS_2_ phototransistor in the OFF and ON states, the photoresponse characteristics were measured by illuminating it with light and fixing it to a gate bias of − 30 and 27 V corresponding to the OFF state and ON state, respectively. Figure 4a shows the change in photocurrent, and Fig. 4b shows the responsivity and specific detectivity according to the drain bias in the OFF state. The specific detectivity is extracted from the equation [26, 33]:

3 $$ {D}^{\ast }=R\cdot {A}^{1/ 2}/{\left(2\cdot q\cdot {I}_{\mathrm{dark}}\right)}^{1/2} $$

where *R* is the responsivity, *A* is the area of the MoS_2_ channel, *q* is the unit charge, and *I*_dark_ is the dark current. In the OFF state, the photocurrent and responsivity increase exponentially with a higher drain bias. Therefore, the photocurrent (responsivity), which was 4.28 × 10^−14^ A (0.12 A/W) when the drain bias was 3 V and light intensity was 10 mW/cm^2^, increased sharply to 1.57 × 10^−8^ A (4.53 A/W) when 27 V drain bias was applied. These results show that the photocurrent and responsivity increase exponentially with the increase in drain bias. On the other hand, in the ON state, the photocurrent (Fig. 4c) and responsivity (Fig. 4d) increase linearly and become saturated as the drain bias increases. When the light intensity is constant at 5 mW/cm^2^ and the drain bias was increased from 3 to 27 V, the photocurrent (responsivity) increased 5-fold from 2.9 × 10^−6^ A (1677 A/W) to 1.5 × 10^−5^ A (8667 A/W). Moreover, the detectivity showed the same tendency as responsivity. In the OFF state (Fig. 4b), it increased from 1.76 × 10^8^ Jones to 2.87 × 10^8^ Jones when the drain bias was increased from 3 to 27 V under a light intensity of 10 mW/cm^2^. In the ON state (Fig. 4d), it increased from 6.14 × 10^9^ Jones to 8.63 × 10^9^ Jones when the drain bias was increased from 3 to 27 V under a light intensity of 5 mW/cm^2^. Therefore, since the diffusion current is dominant in the OFF state, the responsivity increases exponentially as the drain bias increases. On the other hand, the drift current is dominant in the ON state; therefore, the responsivity increases linearly as the drain bias is increased.

**Fig. 5:**
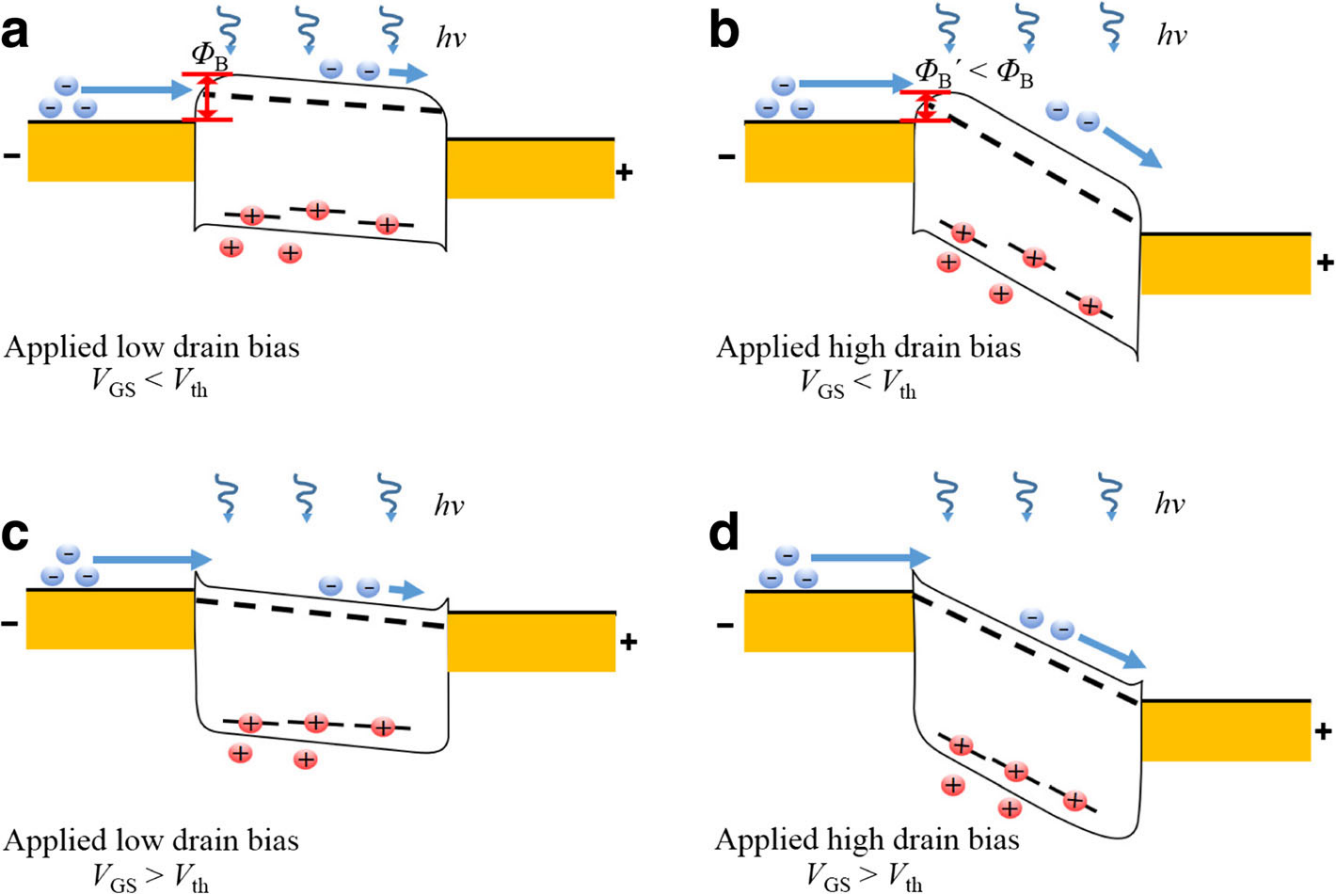
Energy band diagram of multi-layer MoS_2_ phototransistor under illumination at a low-drain bias in the OFF (*V*_GS_ < *V*_th_) state (**a**) and a high-drain bias in the OFF state (**b**). A low-drain bias in the ON (*V*_GS_ > *V*_th_) state (**c**) and a high-drain bias in the ON state (**d**)

The observed drain bias-dependent characteristics of the multi-layer MoS_2_ phototransistor can be explained by the schematic energy band diagram shown in Fig. 5. When the multi-layer MoS_2_ channel is illuminated, the electron-hole pairs are photogenerated in the channel. The photogenerated holes are trapped in the MoS_2_ channel, thus breaking the neutrality of the channel. Then, the positively charged channel attracts more electrons from the source to maintain neutrality, and how much electrons are supplied from the source determines the photoresponse gain. When the applied gate bias is below the threshold, there is a large barrier between the MoS_2_ channel and the source as shown in Fig. 5a and the drain current is limited by the diffusion over the barrier. As the applied drain bias increases (Fig. 5b), the barrier is lowered due to the bending of the MoS_2_ channel, thereby facilitating the supply of electrons for channel neutrality. Therefore, as shown in Fig. 4b, the photoresponsivity improves exponentially for the drain bias. When the applied gate bias is above the threshold, the barrier between MoS_2_ and source is sufficiently low (Fig. 5c), the drain current is limited by the carrier drift in the channel. Therefore, the carrier drift velocity is a major factor in photoresponsivity variation. In this regime, as the applied drain bias increases (Fig. 5d), the carrier velocity and the photoresponsivity linearly increases and saturate at a certain drain bias (~ 10 V) as shown in Fig. 4d.

## Conclusions

We fabricated a multi-layer MoS_2_-based phototransistor and investigated its bias (drain or gate)-controlled photoresponsivity in detail. The change in photoresponsivity according to the bias can be classified into two cases: when the gate bias is smaller than the threshold voltage (OFF state) and when the gate bias is larger than the threshold voltage (ON state). When the gate bias is smaller than the threshold voltage, a small amount of electrons are diffused into the channel, due to large barrier between MoS_2_ and source electrode. As the gate or drain biases increase, the height of the barrier decreases and the number of electrons injected into the channel for neutrality increases. As a result, the photoresponsivity increases exponentially. On the other hand, when the gate bias is greater than the threshold voltage, the photoresponsivity is affected by the carrier velocity rather than the height of barrier because current is limited by carrier drift velocity. As the drain bias increases, the carrier velocity increases linearly and becomes saturated. Therefore, the photoresponsivity increases linearly and becomes saturated. We were able to understand the responsivity variations in multi-layer MoS_2_-based phototransistors according to the gate or drain bias. Thereby, the gain can be controlled to increase the range of application of the MoS_2_ phototransistor and to operate optimally, depending on the purpose and environment.
